# Physiologically Based Pharmacokinetic Modeling to Assess Perpetrator and Victim Cytochrome P450 2C Induction Risk

**DOI:** 10.3390/pharmaceutics17081085

**Published:** 2025-08-21

**Authors:** Marina Slavsky, Aniruddha Sunil Karve, Niresh Hariparsad

**Affiliations:** Drug Metabolism and Pharmacokinetics, Oncology R&D (Research & Development), AstraZeneca, 35 Gatehouse Park Drive, Boston, MA 02451, USA; marina.slavsky@astrazeneca.com (M.S.); aniruddha.karve@astrazeneca.com (A.S.K.)

**Keywords:** CYP2C induction, hepatocytes, PBPK, DDI, pharmacokinetics

## Abstract

**Background:** Accurate assessment of CYP2C induction-mediated drug–drug interactions (DDIs) remains a challenge, despite the importance of CYP2C enzymes in drug metabolism. Limitations in available models and scarce clinical induction data have hampered quantitative preclinical DDI risk evaluation. **Methods:** In this study, the authors utilized an all-human hepatocyte triculture system to capture CYP2C induction using the perpetrators rifampicin, efavirenz, carbamazepine, and apalutamide. In vitro induction parameters were quantified by measuring changes in both mRNA and enzyme activities for CYP2C8, CYP2C9, and CYP2C19. These induction parameters, along with CYP-specific intrinsic clearance (CL_int_) for the victim compounds, were incorporated into a physiologically based pharmacokinetic (PBPK) model, and pharmacokinetics (PK) of known CYP2C substrates were predicted with and without co-administration of perpetrator compounds using clinical dosing regimens. The results were quantitatively compared with the currently utilized mechanistic static modeling (MSM) approach and the reported clinical DDI outcomes. **Results:** By incorporating the measured f_m_ of CYP2C substrates into PBPK modeling, we observed a lower propensity to over- or underpredict the exposure of these substrates as victims of CYP2C induction-based DDIs when co-administered with known perpetrators, which resulted in an excellent correlation to observed clinical outcomes. The MSM approach predicted the CYP3A4 induction-based DDI risk accurately but could not capture CYP2C induction with similar precision. **Conclusions:** Overall, this is the first study that demonstrates the utility of PBPK modeling as a complementary approach to MSM for CYP2C induction-based DDI risk assessment.

## 1. Introduction

Induction of drug metabolizing enzymes can lead to increased metabolism of the drug (autoinduction) or co-administered medications, leading to partial or complete loss of therapeutic effects, which could, therefore, have important consequences in the clinic [1,2,3,4,5,6,7]. In general, in vitro observations from monolayered hepatocyte and two-dimensional sandwich culture models can be quantitatively translated for CYP3A4 using mechanistic static models to assess the clinical induction risk by following the recommendations in the International Council for Harmonization of Technical Requirements for Pharmaceuticals for Human Use (ICH) M12 guidance [8]. However, the same is not true for CYP2C enzymes, even though approximately 20% of clinically utilized drugs are metabolized by CYP2C enzymes, including CYP2C8, CYP2C9, and CYP2C19. Among these substrates of CYP2C enzymes, warfarin, an oral anticoagulant drug, has a narrow therapeutic range, and clinically observed warfarin-associated DDIs can be caused by CYP2C9 induction with aprepitant, carbamazepine, rifampicin, and ritonavir [9,10]. In addition, induction of CYP2C enzymes has been shown to significantly increase the formation of active metabolites of clopidogrel, which may lead to toxicities.

From a perpetrator perspective, while CYP2Cs and CYP3A4 are known to be induced via the activation of the pregnane-X receptor (PXR) and constitutive androstane receptor (CAR), the magnitude of in vitro induction of CYP2C enzymes by rifampicin, in the gold standard monolayered hepatocyte or two-dimensional sandwich cultured models, has a much lower to negligible (<2-fold mRNA fold change in inducer relative to vehicle control) dynamic range compared to CYP3A4 [11,12]. Thus, the recommendation from the IQ Induction Working Group (IWG) was to ascertain the probability of CYP2C induction based on the risk assessment of CYP3A4 induction given that CYP2C enzymes share common regulatory pathways with CYP3A4 [13]. In addition, the IWG concluded that, for successful IVIVe for CYP2C enzymes, alternative in vitro models would be required given that the magnitude of induction in the gold standard hepatocyte models was insufficient in dynamic range [13].

There have been reports of alternative in vitro models to assess the in vitro induction of CYP2C enzymes, with varying levels of success. These include Hepatopac [12] and Hµrel micro-livers [14], both of which are systems in which human hepatocytes are co-cultured with mouse fibroblasts [12,14], as well as a human triculture (HTC) model [15]. The HTC is an all-human, primary cell-based hepatocyte culture model where key structural and functional response elements of mature primary human hepatocytes, including nuclear receptor activation pathways, are expressed and maintained at stable levels for extended periods of time in culture. Given the potential value of the HTC model, the authors identified several clinically relevant CYP2C inducers, such as apalutamide, carbamazepine, efavirenz, and rifampicin (Supplementary Table S1), and generated data in the model that could be utilized to assess perpetrator induction risk.

However, of equal importance when quantifying CYP2C induction risk is incorporation of the fraction metabolized (f_m_) by various metabolic enzymes of the victim drug. Unlike for CYP3A4, there are no selective (f_m_ > 0.9) substrates of CYP2C enzymes [3,16,17,18]. For example, S-warfarin, which is considered to be a prototypical substrate of CYP2C9, has f_m_s of 0.64, 0.05, and 0.12 via CYP2C9, CYP3A4, and CYP2B6, respectively [19]. Similarly, clopidogrel is metabolized by CYP2B6, CYP1A2, and CYP2C19, with f_m_s of 0.06, 0.02, and 0.19, respectively [20]. This limits the utility of static modeling for victim DDI risk assessment, as suggested in the ICH M12 guidance. Another factor that restricts the utility of static modeling is that there are only a limited number of clinical DDI studies specifically designed to assess CYP2C induction, which makes back-translation and use of a correction factor, as is performed for CYP3A4, challenging to provide a quantitative prediction of CYP2C induction risk. Given the aforementioned limitations of MSM, the authors sought to assess the utility of PBPK modeling for the assessment of perpetrator and victim CYP2C induction risk. PBPK modeling serves as a powerful tool for predicting CYP induction-based DDI risk by integrating critical drug-specific and physiological parameters. One of the key advantages of this approach is the ability to incorporate the f_m_ of victim drugs by specific CYP enzymes, which directly influences the magnitude of the interaction. By accounting for the multiple metabolic pathways involved in the clearance of the victim drug, PBPK models enable the mechanistic understanding of the impact of perpetrator-mediated CYP induction on the systemic exposure of the victim drug. This level of granularity enhances the predictive accuracy of DDI risk assessment during early-stage drug development. As such, this approach provided the authors the opportunity to incorporate the f_m_ of victim drugs via multiple enzymes and incorporate the time-variable concentrations of the perpetrator in the liver to predict the effect of CYP induction on the clinical PK of the victim drug [3,21,22]. The in vitro findings and modeling outcomes are discussed in this manuscript.

## 2. Materials and Methods

### 2.1. Selection of Clinical Inducers for In Vitro Characterization

A search of the Certara Drug–Drug Interaction Database (DIDB^™^) identified marketed drugs that demonstrated clinical induction of selective substrates for CYP3A4, CYP2C8, CYP2C9, CYP2C19, or CYP2B6. Clinically relevant concentrations in relation to observed C_max_, solubility, and cytotoxicity were considered when setting incubation concentrations. Three separate human hepatocyte donors were used in the HTC model for the generation of in vitro induction parameters from CYP3A4, CYP2C8, CYP2C9, CYP2C19, and CYP2B6 mRNA and select functional activity changes.

### 2.2. Chemicals and Reagents

Feeder cell thawing medium, human hepatocyte thawing medium, supplemented plating medium, and supplemented culture medium were obtained from LifeNet Health (Virginia Beach, VA, USA). Collagen-I coated 96-well plates, TaqMan^™^ Fast Virus 1-Step Master Mix, and TaqMan^™^ Gene Expression Assay (B2M HS00187842_m1, CYP2C8 HS02383390_s1, CYP2C9 HS02383631_s1, CYP2C19 HS04401150_m1, CYP3A4 Hs00604506_m1, CYP2B6 Hs03044634_m1) were obtained from Thermo Fisher Scientific, (Waltham, MA, USA). RNA isolation kit was obtained from Qiagen (Germantown, MD, USA). Dulbecco′s Phosphate Buffered Saline (DPBS), Penicillin–Streptomycin Solution, ECM Gel from Engelbreth–Holm–Swarm Murine Sarcoma, dimethyl sulfoxide (DMSO), ethanol, acetonitrile, formic acid, phenobarbital, carbamazepine, rifampicin, efavirenz, apalutamide, midazolam, diclofenac, omeprazole, and 5,5-diethyl-1,3-diphenyl-2-iminobarbituric acid were acquired from Millipore Sigma, (St. Louis, MO, USA). All reagents were of sufficient grade or purity.

### 2.3. Preparation of Triculture Model

Human cryopreserved hepatocytes from both male and female donors of differing age and human feeder cells (a mixture of human endothelial and stromal cells) were obtained from LifeNet Health (Virginia Beach, VA, USA) (Supplementary Table S2). The HTC model was constructed according to the vendor’s recommendations. In short, feeder cells were thawed, combined with feeder cell thawing medium prewarmed to 37 °C, gently inverted 4 times, and centrifuged at 400× *g* for 4 min. Feeder cells were resuspended by the addition of 1 mL per vial of supplemented plating medium. Cells were manually counted by trypan blue exclusion and resuspended to a final cell count of 100,000 cells/mL. Feeder cell suspension was added to a collagen-I-coated 96-well plate (100 µL/well) to achieve a final seeding density of 10,000 cells/well and placed in a humidified incubator at 37 °C with 5% CO_2_. The feeder cell attachment was visually inspected 60 min post-plating. Cryopreserved hepatocytes were thawed and combined with human hepatocyte thawing medium prewarmed to 37 °C, followed by centrifugation at 100× *g* for 8 min. Cells were resuspended by the addition of 3 mL per vial of supplemented plating medium, manually counted by trypan blue exclusion, and resuspended to a final cell count of 300,000 cells/mL. Medium from wells containing feeder cells was aspirated, taking care not to disturb the feeder cell layer. Cell suspension was added to a collagen-I-coated 96-well plate (100 µL/well) on top of the feeder cell layer to achieve a final seeding density of 30,000 cells/well. Cells were then placed in a humidified incubator at 37 °C with 5% CO_2_. Cells were allowed to attach for 2 h, followed by aspiration of media to remove any non-adherent hepatocytes and cellular debris. Aspirated media was replaced with 100 µL of prewarmed supplemented culture medium. Supplemented culture medium was replaced daily for the remainder of the 7-day acclimation period.

### 2.4. Determination of Compound Stability in the HTC Model

Stability samples were obtained from the incubations in hepatocyte Donor 1 prior to replacing the dosing solutions every 24 h to determine loss of each test compound over incubation time. Briefly, 30 μL of dosing solution was placed into a 96-well plate containing 60 μL of 100 nM 5,5-diethyl-1,3-diphenyl-2-iminobarbituric acid (internal standard) in acetonitrile with 0.1% formic acid. Samples were further diluted with 120 μL of water containing 0.1% formic acid and centrifuged at 2000× *g* for 10 min, followed by LC–MS/MS analysis. Concentrations obtained were compared with nominal concentrations to determine the extent of loss over the incubation period using Equation 1 under LC–MS/MS conditions described in Supplementary Table S5.

### 2.5. Delineating the CYP Induction Potential of Test Compounds Through Enzyme Activity and mRNA Levels

The DMSO stock solutions of the test compounds apalutamide, carbamazepine, efavirenz, and rifampicin were prepared to generate dosing solutions using an Echo^®^ Acoustic Liquid Handler. Supplemented culture medium (150 µL) was added to the 96-well plate generated by the Echo^®^ Acoustic Liquid Handler to create dosing solutions at various concentrations (final DMSO concentration 0.3%, Supplementary Table S3) in quadruplicate. Hepatocytes were dosed with test compounds, vehicle, or prototypical inducers on day 7 after plating. Supplemented culture medium was aspirated and replaced with fresh media containing test compounds every 24 h. Cell viability was assessed by visual inspection of the monolayer for confluency and morphology. Medium was replaced with fresh medium containing test articles at 24, 48, and 72 h post-initial addition for a total incubation time of 96 h. After 96 h, the cells were washed with 100 µL Dulbecco′s Phosphate Buffered Saline (DPBS). On the last day of the experiment, the cultures were incubated with 100 μL of activity cocktail (Supplementary Table S4) as described in the Supplementary Methods section. Metabolite monitoring was performed for midazolam (1-hydroxy midazolam), omeprazole (5-hydroxy omeprazole), and diclofenac (4-hydroxy diclofenac) as markers for CYP3A4, CYP2C19, and CYP2C9 activity, respectively [23,24,25]. Analyte specific gradient conditions and MS/MS details are presented in Supplementary Table S5.

An assessment of mRNA levels of CYP3A4, CYP2B6, CYP2C8, CYP2C9, and CYP2C19 was performed after activity analysis for CYP3A4, CYP2C9, and CYP2C19. Briefly, the activity cocktail was aspirated, and the cells were washed with 100 µL DPBS and lysed with 100 µL of lysing buffer, followed by mRNA analysis. Both determination of enzyme activity and determination of relative mRNA levels are described in Supplementary Methods.

Derivation of CYP induction parameters was performed using the relative enzyme activity and relative mRNA level changes as previously described [7,26].

### 2.6. Estimation of EC_50_ and E_max_ Parameters from In Vitro Data

In vitro concentration-response data, based on mRNA or activity, were generated for all tested compounds. Each inducer was investigated in a single experiment, with each concentration of inducer evaluated in quadruplicate. Induction parameters E_max_ and EC_50_ were determined by plotting the in vitro fold induction data (mRNA or enzyme activity normalized to the vehicle control) against the nominal in vitro concentration and analyzed using the sigmoidal three parameter model (Equation (2)) in GraphPad Prism (version 10). Induction parameters were determined using nominal concentrations without consideration of the estimated f_u,in vitro_ since the dosing media did not contain albumin and the extent of loss over the incubation period for each inducer was within the analytical error of the experiment (Supplementary Table S3).

### 2.7. Workflow for PBPK and Mechanistic Static Modeling and Statistical Analysis

**Figure 1:**
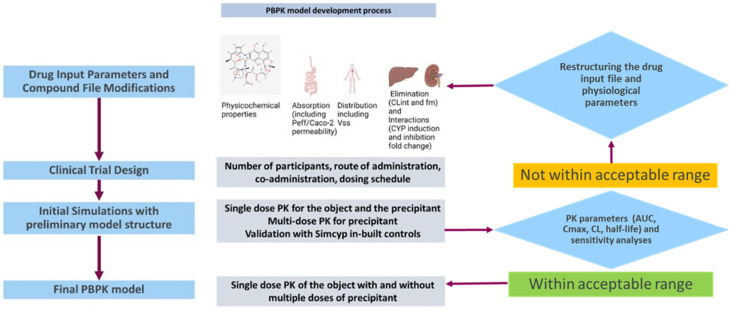
PBPK modeling workflow. The PBPK modeling workflow depicted in the figure involves use of two validation steps: (1) modifying compound files for victim drugs and perpetrator rifampicin with the induction data obtained from the HTC model and validating the model with observed C_max_, AUC, and clearance differences; and (2) re-validating the finalized model by overlaying the observed clinical data and simulated data to finalize the model using the rifampicin–midazolam DDI as a positive control.

Physiologically based pharmacokinetics (PBPK) modeling was performed using Simcyp^®^ v23.1 (industrial named user license was obtained from Certara, UK) simulator to evaluate the estimation of human DDI predictions based on the CYP induction data obtained from HTC model. We utilized available compound library models for sensitive enzyme substrates to determine the PK profile of these compounds administered with and without the inducers rifampicin, apalutamide, carbamazepine, and efavirenz. The induction parameters for the aforementioned inducers were derived from the HTC model, and a standard 10 × 10 trial design was used in Simcyp v.23.1 virtual population library of healthy individuals. The workflow for the PBPK model development is shown in Figure 1.

Midazolam, alfentanil, and atorvastatin, all known CYP3A4-specific substrates, were used as positive controls to ensure model sensitivity. During the sensitivity analyses, a 2-fold change in mRNA induction was used as a representative of the routinely observed variability with the hepatocyte co-culture system, and any changes beyond the 2-fold differences in mRNA induction were considered as significant DDIs. Additionally, the sensitivity analyses included modifying the Cl_int_ values for known CYP enzyme substrates to demonstrate the impact of f_m_ for a specific CYP enzyme on PK predictions. The PK profiles and area under the concentration–time curve (AUC) from the PBPK simulations were compared with the clinically observed AUC changes obtained from DIDB^™^.

The mechanistic static modeling (MSM) was performed by fitting obtained induction parameters into the basic kinetic equation as previously described [26]. The predicted AUCR from the R3 equation calculated according to ICH M12 guidance [8] was compared with the observed clinical data for the clinical dataset presented in Supplementary Table S1.

### 2.8. Equations

The AUC_0–96h_ (AUC_all(Cobs)_) was calculated using the linear trapezoidal rule using Equation (1), and the average concentration over the last day of dosing was calculated by dividing the AUC_0–96h_ by 96 h. The extent of loss was calculated by dividing AUC_0–96h_ by the average calculated dosing concentration.

(1) $${AUC}_{all(Cobs)}=\sum_{t=0}^{n=1}\frac{\left(C_{i}+C_{i+1}\right)\times \left(t_{i+1}-t_{i}\right)}{2}$$

CYP induction parameters were calculated using sigmoidal three-parameter Equation (2):

(2) $$Y=E_{max}/(1\;+\;exp(-(X\;-{EC}_{50})/Hillslope)$$

were Y = relative fold induction, EC_50_ is the concentration eliciting half-maximal induction, and E_max_ is the maximum fold induction.

AUCR was calculated using Equation (3):

(3) $$R3=\frac{1}{1+d\times \;\frac{E_{max}\times \;E_{max}\;\times 10}{E_{max}+\;E_{max}\;\times 10}\;\;}$$

## 3. Results

### 3.1. Induction of CYPs in HTC

The depletion of test articles was monitored over the 24 h dosing intervals in one human hepatocyte donor. The CYP induction parameters EC_50_ and Emax were determined using nominal concentrations without adjusting for the estimated f_u,in vitro_ as the dosing media did not contain albumin and the extent of compound loss during the incubation period for each inducer was within the analytical error of the experiment (Supplementary Table S3).

The induction parameters derived using the HTC model are detailed in Table 1 and Table 2.

All tested compounds demonstrated concentration-dependent increases in relative fold mRNA levels for CYP3A4, CYP2C19, CYP2C8, CYP2C9, and CYP2B6 and in relative enzyme activity for CYP3A4 and CYP2C19.

**Figure 2:**
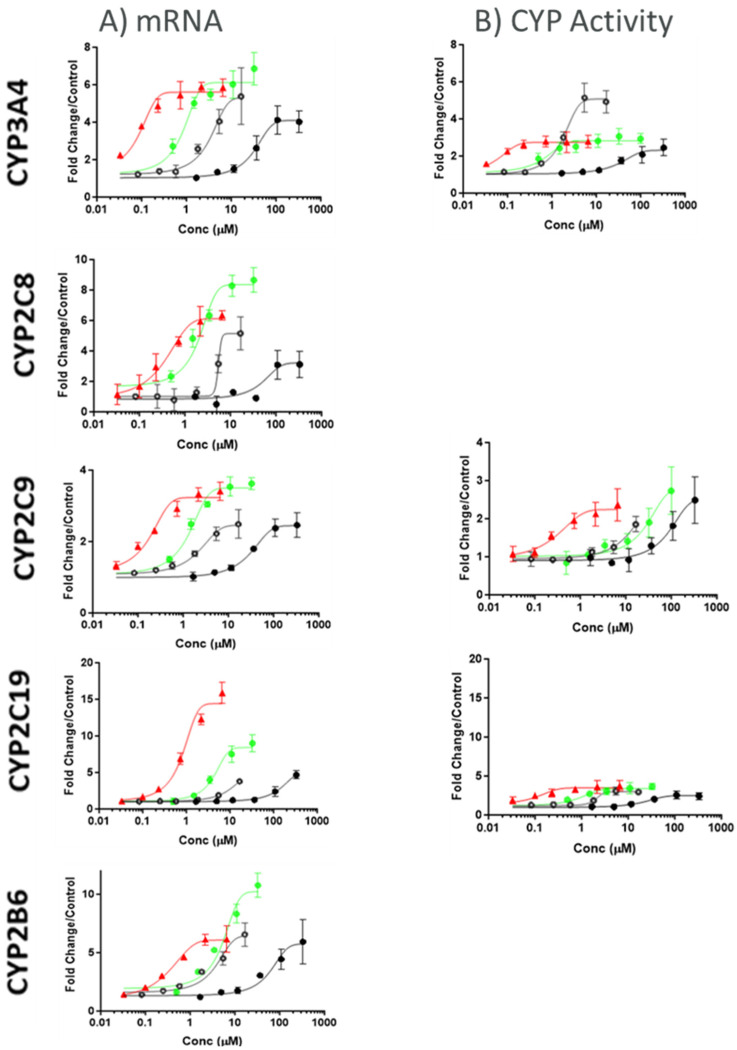
Representative concentration response graphs from donor 1 for HTC model ((**A**)—mRNA, (**B**)—CYP activity). Carbamazepine in closed black circles, rifampicin in closed red triangles, efavirenz in open black circles, apalutamide in closed green circles. The fold change in mRNA level compared to the vehicle control value is displayed on the *y*-axis, with nominal added concentrations displayed on the *x*-axis. Values represent mean ± SD of n = 4 values.

As shown in Figure 2 and Table 1, the EC_50_ values were lowest with rifampicin for CYP3A4 (0.066–0.128 µM), CYP2C19 (0.803–1.64 µM), CYP2C8 (0.260–0.504 µM), CYP2C9 (0.0874–0.149 µM), and CYP2B6 (0.399–0.53 µM) and highest fold-induction (E_max_) for CYP2C19 (8.45–19.9) as observed in all of the donors. On the other hand, the highest fold-induction (E_max_) for CYP3A4 (6.12–20.8), CYP2C8 (4.55–8.36), CYP2C9 (2.73–3.63), and CYP2B6 (6.71–10.2) was observed after treatment with apalutamide across all the donors.

Based on the one-way ANOVA to compare E_max_ differences across perpetrators, apalutamide and rifampicin were observed to have no statistically significant differences between the observed E_max_ values, but the E_max_ values for efavirenz and carbamazepine were significantly different from each other and also differed significantly from the E_max_ of apalutamide and rifampicin.

Similar analyses performed for EC_50_ revealed that the EC_50_ values for all four perpetrators had a statistically significant difference, with a *p*-value < 0.05. The magnitude of CYP3A4 induction for donor 2 (mRNA) and donor 3 (CYP activity) was the highest among the three donors for all tested clinical inducers. The magnitude of CYP2B6 induction for donor 3 (mRNA) was the highest among the three donors for all tested clinical inducers. The magnitude of CYP2C19 induction by mRNA and CYP activity for donor 3 (mRNA) was the highest among the three donors for all tested clinical inducers. The magnitude of CYP2C9 induction for donor 1 was the highest among the three donors for all tested clinical inducers. However, the relative enzyme activity of CYP2C9 remained at or near 2-fold. The magnitude of CYP2C8 induction by mRNA for donor 1 and donor 3 was the highest among the three donors for all tested clinical inducers.

### 3.2. Comparison of CYP Induction-Mediated DDI Risk Assessment Using Mechanistic Static Modeling Approaches (MSM) and PBPK

The CYP induction MSM assessment was based on the experimentally determined in vitro EC_50_ and the maximum fold-induction (E_max_) for rifampicin, apalutamide, efavirenz, and carbamazepine. The R3 equation along with the EC_50,_ and E_max_ for the perpetrators and the recommended scaling factor (d) of 1 and a correction factor of 10X for CYP3A4 (ICH M12 Guidance) were used to predict the AUC ratios. For PBPK modeling, additional sensitivity analyses were performed for the perpetrators comparing the clinical PK of the perpetrators with the PBPK-predicted PK profiles at steady-state. The AUCs for all of the perpetrators were found to be within 0.8–1.25-fold of the clinically observed AUC at steady-state. Furthermore, additional sensitivity analyses for the induction potential of the perpetrators were performed using midazolam as a control. This involved changing the E_max_ and EC_50_ values for the perpetrators in the PBPK model. The data obtained during these sensitivity analyses indicated that increasing the E_max_ of the perpetrators for CYP3A4 by 2-fold and decreasing the EC_50_ of the perpetrators for CYP3A4 by 2-fold resulted in a significant decrease in the AUC ratios of midazolam (0.80), further validating the efficiency of the PBPK model in predicting CYP induction. In addition to the perpetrators, the sensitivity analyses for the victim drugs were performed by changing the CL_int_ of the victim drugs (used as a surrogate for f_m_CYP), which also resulted in significant changes in the AUC ratios of the victims.

**Figure 3:**
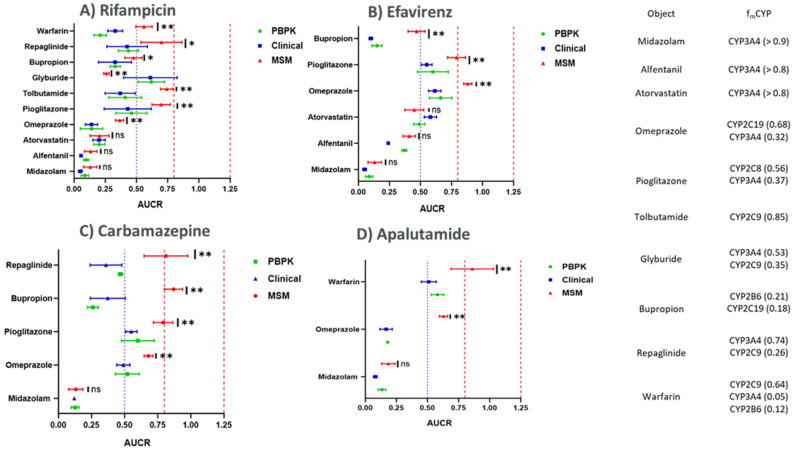
Prediction of CYP induction with HTC data using PBPK vs. static modeling after treatment with (**A**) rifampicin, (**B**) efavirenz, (**C**) carbamazepine and (**D**) apalutamide. AUC ratios of victim drugs with and without rifampicin are shown as green circles denoting PBPK simulated data AUC ratios, red circles denoting MSM predicted AUC ratios, and blue circles denoting clinically observed AUC ratios for the victim drugs. Victim drug f_m_ values for each CYP involved in their metabolism are listed below the graph. The dashed red lines indicate the acceptable average bioequivalence range (0.8–1.25), and the dotted blue lines indicate the non-significant DDI range (0.5–2.0). AUC ratio values are represented on the *y*-axis as mean ± SD (n.s. = Not significant; * = *p*-value < 0.01; ** = *p*-value < 0.005).

The details for clinically observed DDI data and the clinical trial design used for modeling are shown in Table 3, and the data obtained from PBPK simulations using CYP induction EC_50_ and E_max_ from the HTC model are presented in Figure 3 and Table 4.

The predicted AUC ratios for the victim drugs with and without perpetrator-mediated CYP induction were compared with the AUC ratios derived using MSM and the clinically observed AUC ratios for the victim drugs obtained from Certara DIDB^TM^. Rifampicin was found to be the most significant inducer for CYP3A4, CYP3A5, CYP2C19, CYP2C8, CYP2C9, and CYP2B6 enzymes at clinically relevant concentrations. The PBPK-derived AUC ratios for the controls were observed to range between 0.08 and 0.13 for midazolam, 0.09 and 0.13 for alfentanil, and 0.20 and 0.27 for atorvastatin. The clinical and MSM-derived AUC ratios for the controls were not significantly different from the PBPK-derived AUC ratios as highlighted in Figure 3.

On the other hand, for the other victim drugs used in this study as markers for CYP2C19-, CYP2C8-, CYP2C9-, and CYP2B6-mediated clearance, the PBPK-predicted AUC ratios ranged from 0.05 to 0.23 for omeprazole, 0.16 to 0.26 for warfarin, 0.34 to 0.58 for pioglitazone, 0.28 to 0.53 for tolbutamide, 0.5 to 0.7 for glyburide, 0.35 to 0.51 for repaglinide, and 0.27 to 0.37 for bupropion. In comparison, the AUC ratios obtained using the MSM approach were as follows: 0.33–0.39 for omeprazole, 0.51–0.62 for warfarin, 0.62–0.76 for pioglitazone, 0.69–0.79 for tolbutamide, 0.23–0.27 for glyburide, 0.54–0.86 for repaglinide, and 0.42–0.54 for bupropion. The MSM-predicted AUC ratios for the victim drugs of CYP2C19, CYP2C8, CYP2C9, and CYP2B6 differed significantly from the clinical observations and the PBPK-predicted AUC ratios, as shown in Figure 3. There was no statistically significant difference observed between PBPK-derived AUC ratios and the clinically observed AUC ratios when analyzed using one-way ANOVA.

**Figure 4:**
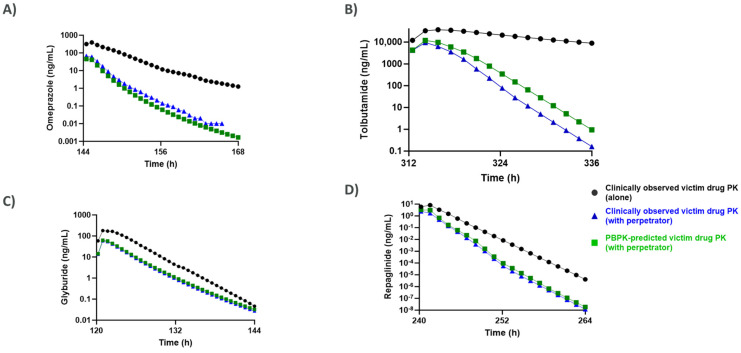
PBPK−predicted PK profiles of victim drugs (**A**) omeprazole, (**B**) tolbutamide, (**C**) glyburide, and (**D**) repaglinide, administered with/without rifampicin, overlayed with the clinically observed PK profiles obtained from DIDB^TM^.

Unlike the MSM approach, the PBPK modeling also enabled prediction of concentration–time curves for the victim drugs with and without perpetrator administration, which were overlayed with the clinically observed PK profiles of the victim drugs co-administered with rifampicin as shown in Figure 4.

No statistically significant difference (*p*-value > 0.05) was observed between the PK profiles for the victim drugs when compared with the clinically observed PK profiles of the victims after administration with the perpetrator when analyzed using one-way ANOVA to determine statistical significance. The authors used rifampicin as the perpetrator for generating complete PK profiles of victim drugs, as it was observed to have the most significant induction effects at clinically relevant concentrations and had extensive availability of clinical DDI data for all the victim drugs tested during this study. The f_m_ values and %AUC reduction for the test compounds after administration with rifampicin are shown in Table 4, and the AUC ratios obtained using other perpetrators are shown in Figure 3.

## 4. Discussion

The necessity for dedicated clinical DDI studies is often informed by leveraging in vitro data that is generated preclinically. The ICH M12 DDI guideline suggests that the potential of a new chemical entity (NCE) to induce hepatic enzymes via activation of nuclear receptors, such as PXR, CAR, and AhR (aryl hydrocarbon receptor) and, if relevant, other drug regulation pathways, should be evaluated for DDI risk if there is robust CYP3A4 induction [3]. Given that CYP3A4 shares transcriptional regulation factors with CYP2C isoforms, it has been suggested that it may be possible to ascertain the risk of CYP2C induction based on the assessment for CYP3A4 induction using static and dynamic models [45]. This is not always possible, however, given that ~70% of compounds can be inducers as well as time-dependent inhibitors of CYP3A4 [8]. An additional limitation for the IVIVe of non-CYP3A enzymes, such as CYP2C, is the historically low dynamic range as well as inconsistent and contradictory data using standard hepatocyte monoculture systems [3,12,13,30,46,47,48,49].

Given the challenges observed with the assessment of CYP2C induction using the gold standard monoculture systems, Dixit et al., 2016 [12], attempted to conduct an evaluation using the micropatterned co-culture system Hepatopac to determine if a higher dynamic range and reproducible CYP2C induction can be obtained in a more physiologically relevant system. A higher dynamic range was observed for CYP2C8, CYP2C9, and CYP3A4 in the Hepatopac compared to the monoculture system. However, the authors did not generate CYP2C19 induction data [50]. In another study, Aluri et al., 2024 [14], utilized Hµrel micro-livers to assess the CYP induction potential of aminobenzotriazole. The study included rifampicin as a control perpetrator, where the authors observed robust CYP3A4, CYP1A2, CYP2C8, CYP2C9, and CYP2B6 induction in Hµrel micro-livers after treatment with rifampicin [14]. Among the other ongoing efforts to evaluate CYP2C induction using in vitro systems, Ref. [9] evaluated CYP induction using a 3D-spheroid culture of primary human hepatocytes (PHH). The authors reported robust induction of CYP3A4, with a 6-fold increase in CYP3A4 mRNA levels, in the PHH spheroids after treatment with rifampicin. The authors also observed induction of CYP2B6 (9-fold), CYP2C8 (12-fold), and CYP2C9 (2-fold) mRNA levels. CYP2C19 induction (8-fold) was observed in two of the three donors, with one of the donors demonstrating no changes in CYP2C19 mRNA or protein levels after treatment with rifampicin [12]. More recently, Ramsden et al. utilized the HTC model to evaluate the net induction/inhibition-based DDI risk for a variety of perpetrators of CYP3A4, CYP2C8, CYP2C9, and CYP2C19 [15].

Another important consideration for the in vitro assessment of CYP induction is the variability in CYP expression and inducibility between hepatocyte donors. These differences stem from a range of genetic, epigenetic, and environmental factors, including polymorphisms in nuclear receptors, such as PXR and CAR, variable exposure histories to xenobiotics, and hepatic disease status [1,51]. For instance, rifampicin induces CYP3A4 in a highly donor-dependent manner, with some donors showing robust induction (>10-fold) while others display muted responses [4]. This heterogeneity complicates the extrapolation of in vitro induction potential to clinical risk without mechanistic modeling approaches that account for population variability.

A key step after generating in vitro data is translating this information into meaningful clinical context. While substantial clinical data exist for perpetrator drugs and sensitive CYP3A4 substrates, such as midazolam, alfentanil, and atorvastatin, this is not the case for CYP2C enzymes, where available substrates are typically metabolized by multiple CYP enzymes (Table 3). This multifaceted metabolism poses challenges for static modeling approaches, including the relative induction score (RIS), by complicating the process of assigning risk to a single metabolic pathway.

It is important to acknowledge, however, that mechanistic static modeling serves as a valuable and widely adopted tool, particularly when extensive clinical or in vivo data are available. MSM approaches, with their reliance on parameters such as maximum unbound plasma concentrations and standardized correction factors (e.g., ten times unbound Cmax for each two-fold increase in CYP3A4 mRNA, as described by Ramsden et al., 2025) [15], provide a practical framework for an initial, high-throughput assessment of perpetrator DDI risk. Their straightforward equations and relatively low computational burden make them readily accessible for early-stage screening or for scenarios involving well-characterized CYP enzymes and substrates.

Nonetheless, static models inherently assume a constant concentration of the inducer throughout the course of administration and may not fully capture the nuances of temporal changes in CYP expression or drug exposure [52]. This can be especially limiting when applied to CYP2C-mediated DDIs, where both victim and perpetrator compounds may display variable pharmacokinetics and dynamic changes over time.

In light of these considerations, we chose to utilize physiologically based pharmacokinetic (PBPK) modeling in this study to further refine DDI risk assessment. PBPK models offer a notable advantage over static approaches in their ability to simulate systemic concentrations of the perpetrator across different dosing regimens and to dynamically incorporate time-varying changes in drug and enzyme levels. By integrating in vitro induction parameters derived from the HTC system into the PBPK model, we observed improved prediction accuracy for the pharmacokinetics of victim drugs—as reflected in Figure 3 and Table 4—compared to the MSM approach.

Specifically, prediction of C_max_ and AUC changes for rifampicin using the PBPK model were consistently within the single standard deviation range of clinically observed outcomes for drugs with significant CYP2C involvement. Substrate-specific AUC ratios generated from PBPK simulations aligned closely with clinical data, even for compounds metabolized by multiple enzymes (such as omeprazole, tolbutamide, pioglitazone, glyburide, bupropion, and repaglinide), whereas the MSM often did not accurately recapitulate these patterns. However, it is worth noting that, for sensitive CYP3A4 substrates, both PBPK and MSM approaches produced accurate predictions, underlining the enduring utility of MSM in these contexts. The PBPK approach also enabled detailed prediction of the entire concentration–time curve for victim drugs, providing valuable insight into the temporal profile of DDI and permitting direct comparison with observed clinical data (Figure 4). Additionally, sensitivity analyses conducted by varying enzyme clearance parameters further highlighted the importance of accurate fmCYP estimation in DDI risk prediction—precision that was well-supported by the PBPK modeling strategy. The ability to dynamically predict both perpetrator and victim concentrations, particularly within the liver, adds a clinically relevant dimension that complements the strengths of MSM.

In summary, incorporating in vitro-derived induction parameters for CYP2C8, CYP2C9, CYP2C19, and CYP3A4 into PBPK modeling resulted in quantitative and clinically concordant predictions of drug interactions, suggesting that such approaches may help reduce dependence on dedicated clinical DDI studies. While mechanistic static models continue to provide valuable preliminary insights, especially for well-characterized interactions or for quick assessments, integrating novel in vitro tools and PBPK modeling facilitates a more comprehensive risk assessment for both perpetrator and victim CYP2C induction, particularly in complex scenarios involving multiple metabolic pathways.

## Figures and Tables

**Table 1:** Summary of nominal derived induction parameters from mRNA analysis.

Test Article	Hepatocyte Donor Designation					
	Donor 1		Donor 2		Donor 3	
	EC_50_ (μM)	E_max_ Fold	EC_50_ (μM)	E_max_ Fold	EC_50_ (μM)	E_max_ Fold
	**CYP3A4**					
Apalutamide	0.690	6.12	1.61	20.8	1.08	7.45
Carbamazepine	23.6	4.09	61.2	9.93	28.5	3.53
Efavirenz	2.61	5.32	4.38	11.3	3.04	5.31
Rifampicin	0.0655	5.60	0.128	13.4	0.0602	4.87
	**CYP2C8**					
Apalutamide	1.68	8.36	1.22	4.55	1.26	7.20
Carbamazepine	35.6	3.21	20.0	2.26	40.2	5.07
Efavirenz	4.48	5.22	1.97	2.85	2.95	5.11
Rifampicin	0.504	6.89	0.260	4.51	0.307	7.36
	**CYP2C9**					
Apalutamide	0.794	3.50	0.419	3.63	0.474	2.73
Carbamazepine	9.86	2.44	NC	<2	NC	<2
Efavirenz	0.455	2.46	NC	<2	NC	<2
Rifampicin	0.0876	3.23	0.0784	2.87	0.149	2.57
	**CYP2C19**					
Apalutamide	4.01	8.42	5.22	7.70	6.04	14.6
Carbamazepine	124	5.11	186	4.48	182	7.22
Efavirenz	8.30	4.79	NC	<2	2.91	3.56
Rifampicin	1.64	19.9	0.803	8.45	1.47	17.9
	**CYP2B6**					
Apalutamide	4.44	10.2	2.98	6.71	2.96	9.06
Carbamazepine	46.9	5.74	41.9	4.84	59.0	6.93
Efavirenz	2.76	6.46	3.67	6.61	4.01	9.44
Rifampicin	0.415	6.71	0.53	5.91	0.399	8.44

**Table 2:** Summary of nominal derived induction parameters from activity analysis.

Test Article	Hepatocyte Donor Designation					
	Donor 1		Donor 2		Donor 3	
	EC_50_ (μM)	E_max_ Fold	EC_50_ (μM)	E_max_ Fold	EC_50_ (μM)	E_max_ Fold
	**CYP3A4**					
Apalutamide	0.686	2.96	0.709	3.03	1.50	10.6
Carbamazepine	6.40	2.31	2.70	3.38	17.8	4.44
Efavirenz	1.43	5.07	0.259	2.79	4.54	16.3
Rifampicin	0.0509	2.84	0.0652	3.45	0.0897	7.74
	**CYP2C9**					
Apalutamide	13.1	2.79	NC	<2	23.7	3.25
Carbamazepine	41.4	2.52	NC	<2	NC	<2
Efavirenz	4.29	2.40	NC	<2	NC	<2
Rifampicin	0.469	2.44	NC	<2	NC	<2
	**CYP2C19**					
Apalutamide	0.455	3.38	1.03	3.62	0.865	9.66
Carbamazepine	8.14	2.48	2.05	3.63	18	4.54
Efavirenz	0.946	3.04	0.297	3.52	2.48	6.87
Rifampicin	0.0438	3.48	0.0816	4.09	0.105	9.32

**Table 3:** Clinical trial design, including dosing regimens for victims and perpetrators used for PBPK modeling of CYP induction.

Perpetrator	Perpetrator Dose	Object	Object Dose	Object Metabolism CYP (f_m_)	References
Rifampicin	600 mg (5 days)	Midazolam	3 mg (after rifampicin on day 5)	CYP3A4 (>0.9)	[27,28]
	600 mg (5 days)	Alfentanil	4 mg (after rifampicin on day 5)	CYP3A4 (≥0.8)	[27,29]
	600 mg (5 days)	Atorvastatin (acid)	40 mg (after rifampicin on day 5)	CYP3A4 (>0.9)	[30,31]
	450 mg (6 days)	Omeprazole	20 mg (after rifampicin on day 6)	CYP2C19 (0.68) CYP3A4 (0.32)	[32,33]
	600 mg (6 days)	Pioglitazone	30 mg (after rifampicin on day 5)	CYP2C8 (0.56) CYP3A4 (0.37)	[34]
	600 mg (14 days)	Tolbutamide	500 mg (after rifampicin on day 14)	CYP2C9 (0.85) Other CYPs (0.15)	[35,36]
	600 mg (5 days)	Glyburide	1.75 mg (12.5 h after final rifampicin dose)	CYP3A4 (0.53) CYP2C9 (0.35) CYP2C8 (0.12)	[37,38]
	600 mg (10 days in the evening)	Bupropion	150 mg (on day 8 of rifampicin dosing)	CYP2B6 (0.21) CYP2C19 (0.18)	[39,40]
	600 mg (10 days)	Repaglinide	0.5 mg (12.5 h after final rifampicin dose)	CYP3A4 (0.74) CYP2C8 (0.26)	[41,42,43]
	600 mg (3 days)	Warfarin	1.5 mg/kg (on day 4 after rifampicin dose)	CYP2C9 (0.64) CYP3A4 (0.05) CYP2B6 (0.12)	[44]

**Table 4:** Comparison of percent reduction in AUC of victim drugs obtained using PBPK, MSM, and clinically observed data.

Substrate	Substrate Metabolism CYP (f_m_)	% AUC Reduction (PBPK Modeling)	% AUC Reduction (Mechanistic Static Modeling)	% AUC Reduction (Clinically Observed)	References
Midazolam	CYP3A4 (>0.9)	91.4	87.1	94.7	[27,28]
Alfentanil	CYP3A4 (≥0.8)	90.3	86.7	94.5	[29]
Atorvastatin (acid)	CYP3A4 (>0.8)	79.9	79.6	80.2	[30]
Omeprazole	CYP2C19 (0.68) CYP3A4 (0.32)	86.4	64.2	86.1	[32,33]
Pioglitazone	CYP2C8 (0.56) CYP3A4 (0.37)	54.03	31.4	56.3	[34]
Tolbutamide	CYP2C9 (0.85) Other CYPs (0.15)	59.8	25.8	63.5	[35,36]
Glyburide	CYP3A4 (0.53) CYP2C9 (0.35) CYP2C8 (0.12)	38.2	74.1	38.9	[37,38]
Bupropion	CYP2B6 (0.21) CYP2C19 (0.18)	67.6	53.4	67.2	[39,40]
Repaglinide	CYP3A4 (0.74) CYP2C8 (0.26)	56.5	31.4	57.5	[41,42,43]

## Data Availability

The data that support the findings of this study are available from the corresponding author upon reasonable request. Some data may not be made available because of privacy or ethical restrictions.

## Supplementary Materials

The following supporting information can be downloaded at: https://www.mdpi.com/article/10.3390/pharmaceutics17081085/s1, Table S1. Clinical inducers tested in this study, Table S2. Donor demographics, Table S3. Summary of stability results from Donor 2017942-01 following 96-h treatment, Table S4. Activity Cocktail, Table S5. LC/MS/MS conditions for enzyme activity and metabolic stability, and supplementary methods.
