# Supplementary material for: Physiologically Based Pharmacokinetic Modeling to Assess Perpetrator and Victim Cytochrome P450 2C Induction Risk
Source: Pharmaceutics. 2025 Aug 21;17(8):1085. doi: 10.3390/pharmaceutics17081085 (PMC12389355; doi:10.3390/pharmaceutics17081085)
Supplement: Supplementary file 1 [file pharmaceutics-17-01085-s001.zip › pharmaceutics-3703012-supplementary.pptx]

## Slide 1
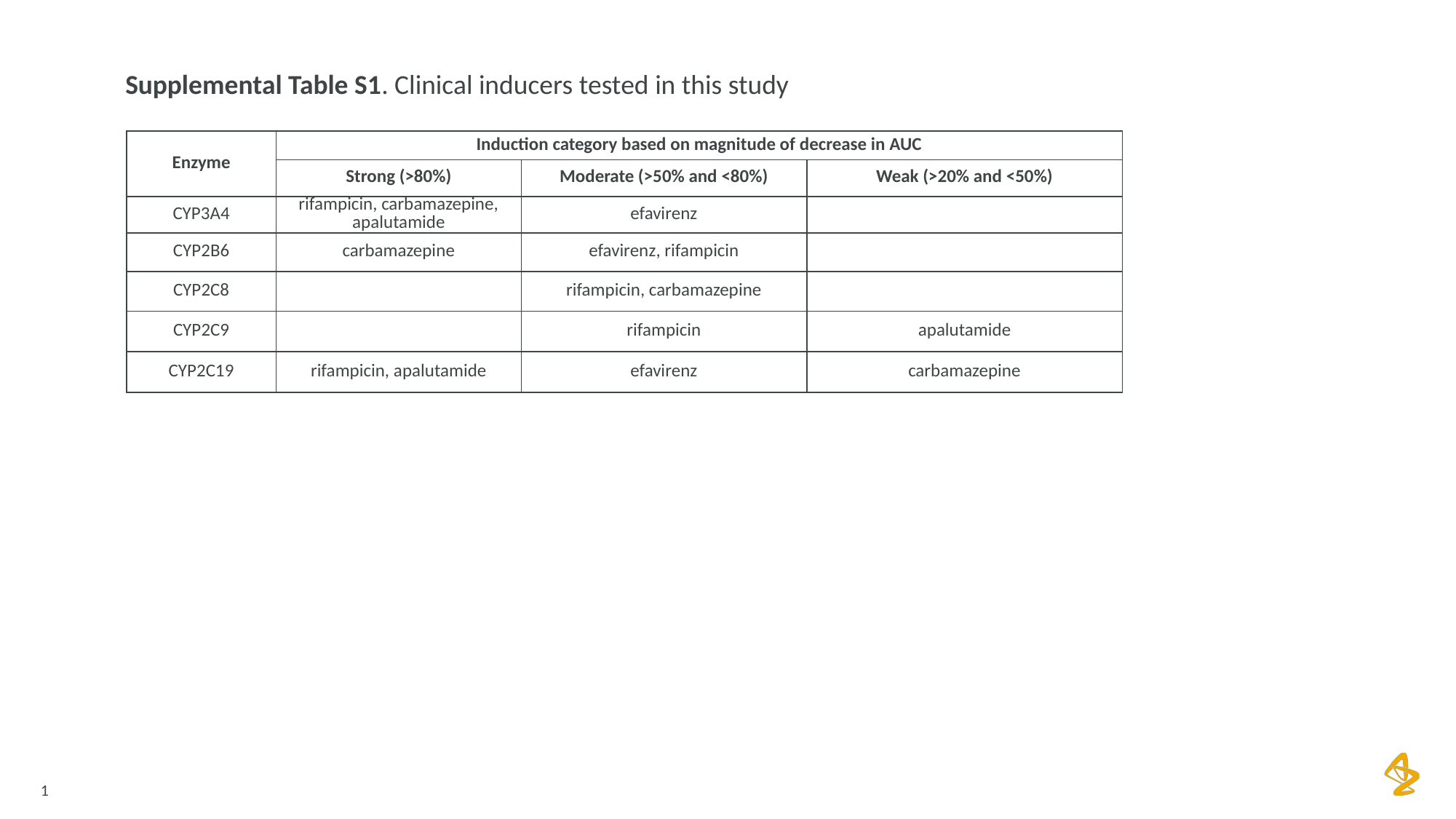

Supplemental Table S1. Clinical inducers tested in this study
| Enzyme | Induction category based on magnitude of decrease in AUC | | |
| --- | --- | --- | --- |
| | Strong (>80%) | Moderate (>50% and <80%) | Weak (>20% and <50%) |
| CYP3A4 | rifampicin, carbamazepine, apalutamide | efavirenz | |
| CYP2B6 | carbamazepine | efavirenz, rifampicin | |
| CYP2C8 | | rifampicin, carbamazepine | |
| CYP2C9 | | rifampicin | apalutamide |
| CYP2C19 | rifampicin, apalutamide | efavirenz | carbamazepine |
1

## Slide 2
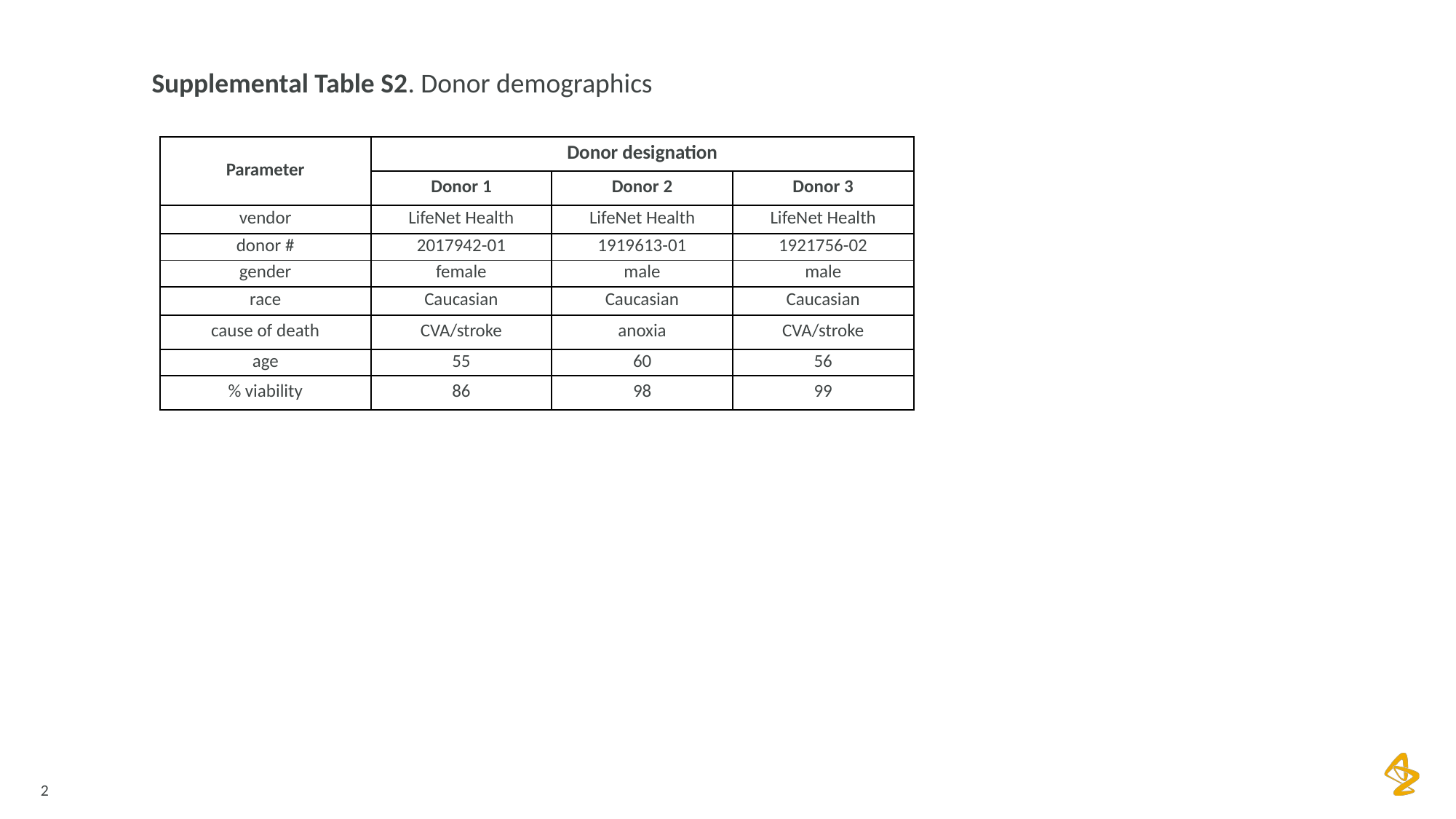

Supplemental Table S2. Donor demographics
| Parameter | Donor designation | | |
| --- | --- | --- | --- |
| | Donor 1 | Donor 2 | Donor 3 |
| vendor | LifeNet Health | LifeNet Health | LifeNet Health |
| donor # | 2017942-01 | 1919613-01 | 1921756-02 |
| gender | female | male | male |
| race | Caucasian | Caucasian | Caucasian |
| cause of death | CVA/stroke | anoxia | CVA/stroke |
| age | 55 | 60 | 56 |
| % viability | 86 | 98 | 99 |
2

## Slide 3
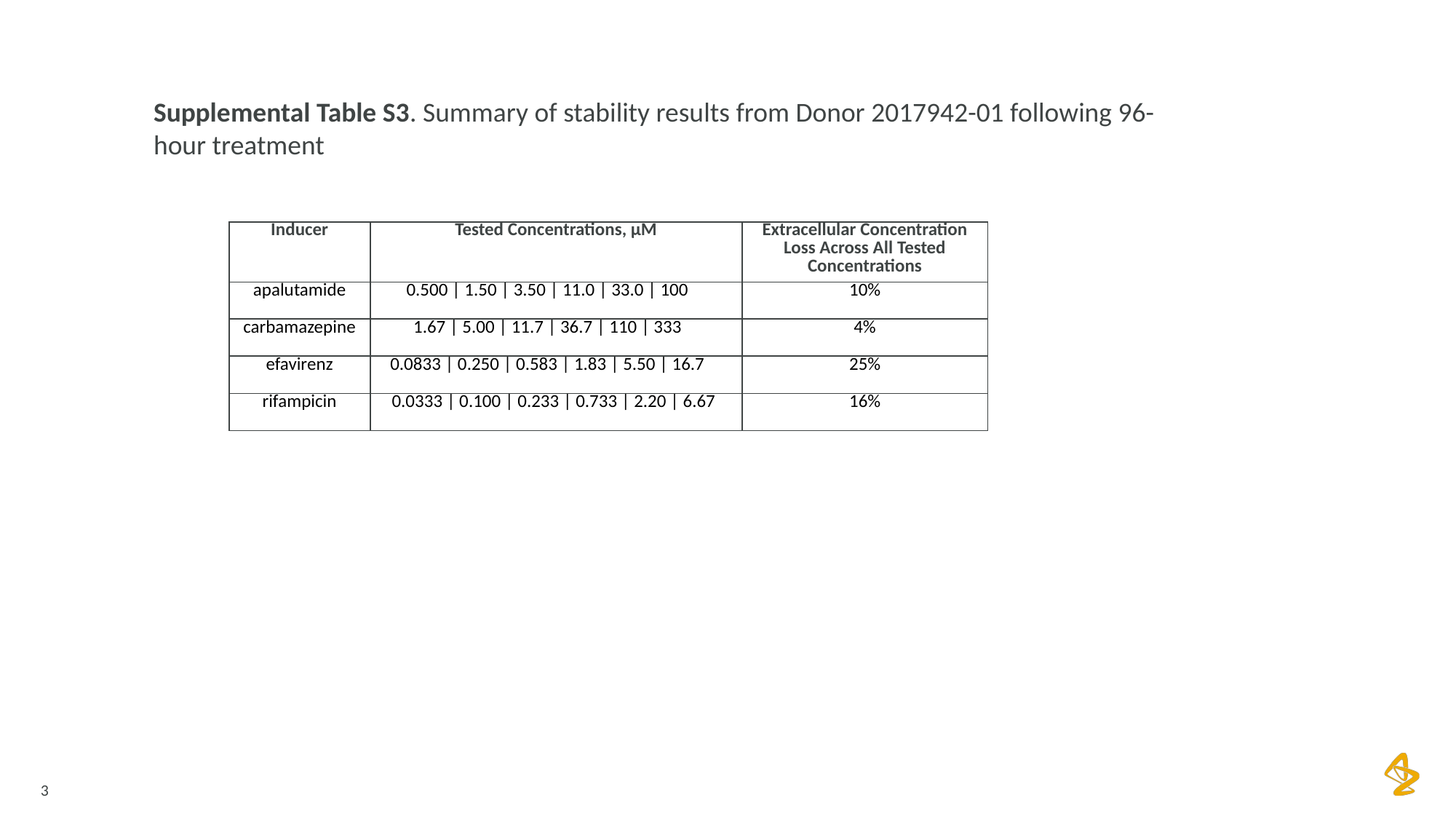

Supplemental Table S3. Summary of stability results from Donor 2017942-01 following 96-hour treatment
| Inducer | Tested Concentrations, µM | Extracellular Concentration Loss Across All Tested Concentrations |
| --- | --- | --- |
| apalutamide | 0.500 | 1.50 | 3.50 | 11.0 | 33.0 | 100 | 10% |
| carbamazepine | 1.67 | 5.00 | 11.7 | 36.7 | 110 | 333 | 4% |
| efavirenz | 0.0833 | 0.250 | 0.583 | 1.83 | 5.50 | 16.7 | 25% |
| rifampicin | 0.0333 | 0.100 | 0.233 | 0.733 | 2.20 | 6.67 | 16% |
3

## Slide 4
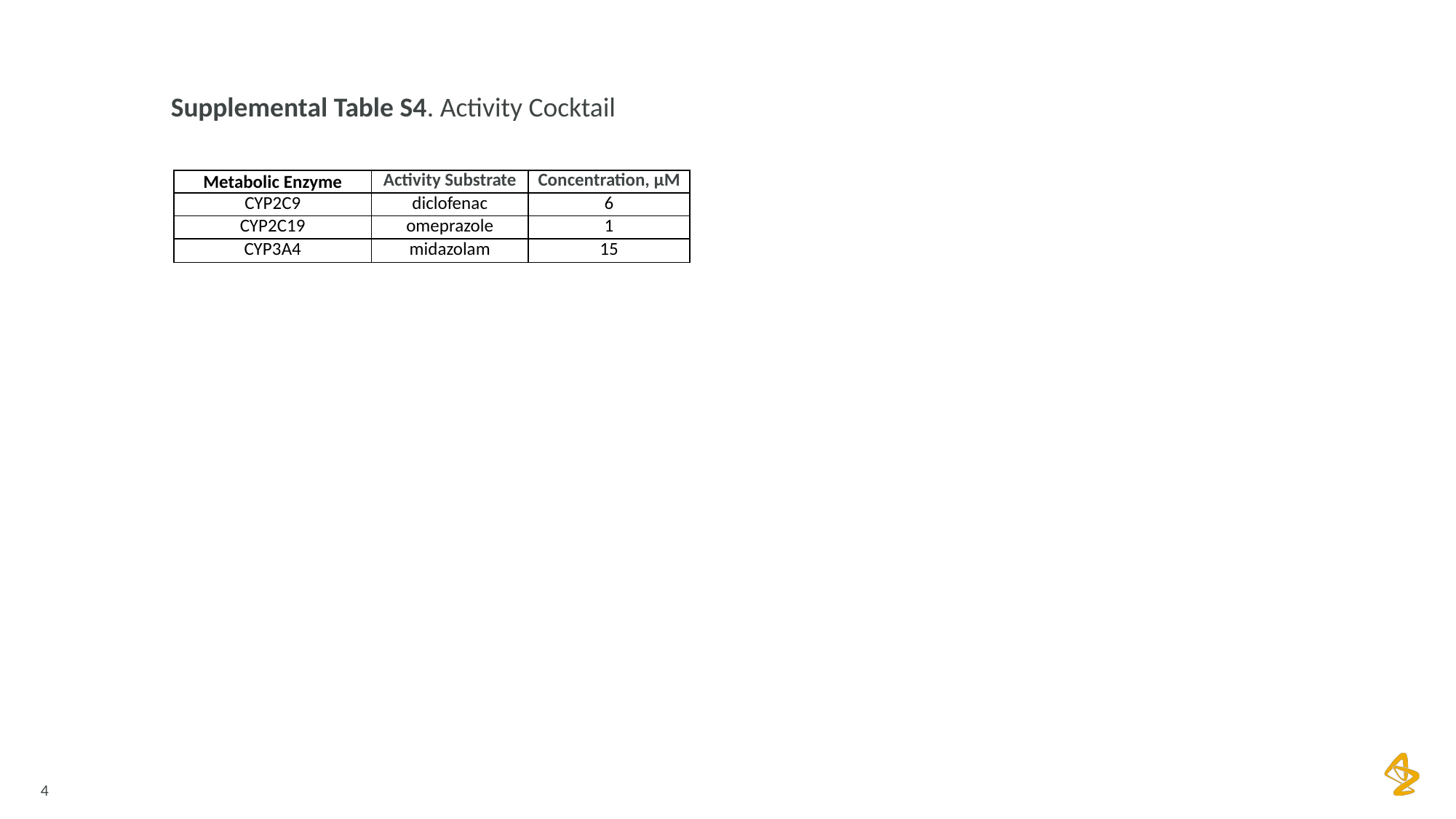

Supplemental Table S4. Activity Cocktail
| Metabolic Enzyme | Activity Substrate | Concentration, µM |
| --- | --- | --- |
| CYP2C9 | diclofenac | 6 |
| CYP2C19 | omeprazole | 1 |
| CYP3A4 | midazolam | 15 |
4

## Slide 5
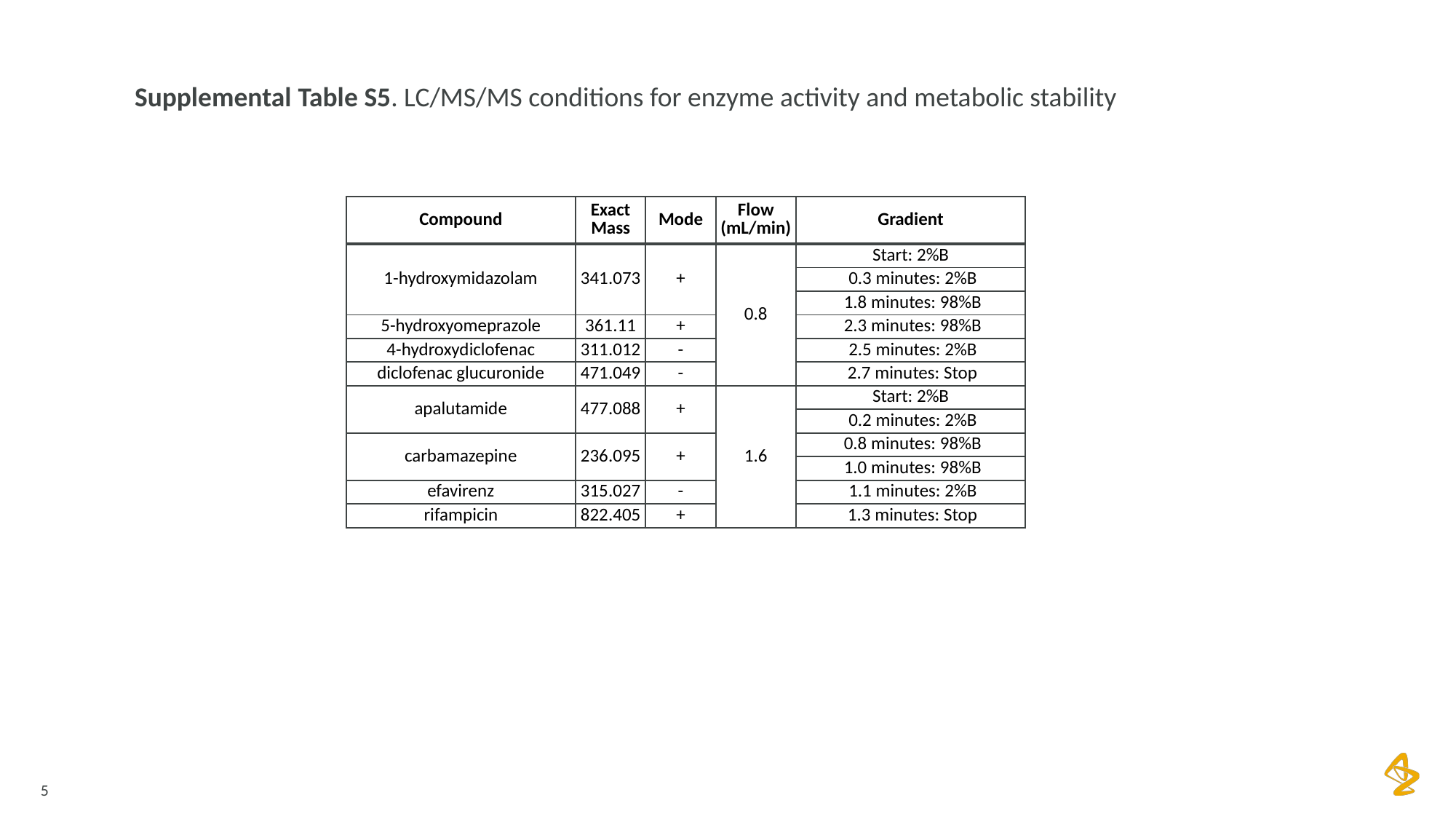

Supplemental Table S5. LC/MS/MS conditions for enzyme activity and metabolic stability
| Compound | Exact Mass | Mode | Flow (mL/min) | Gradient |
| --- | --- | --- | --- | --- |
| 1-hydroxymidazolam | 341.073 | + | 0.8 | Start: 2%B |
| | | | | 0.3 minutes: 2%B |
| | | | | 1.8 minutes: 98%B |
| 5-hydroxyomeprazole | 361.11 | + | | 2.3 minutes: 98%B |
| 4-hydroxydiclofenac | 311.012 | - | | 2.5 minutes: 2%B |
| diclofenac glucuronide | 471.049 | - | | 2.7 minutes: Stop |
| apalutamide | 477.088 | + | 1.6 | Start: 2%B |
| | | | | 0.2 minutes: 2%B |
| carbamazepine | 236.095 | + | | 0.8 minutes: 98%B |
| | | | | 1.0 minutes: 98%B |
| efavirenz | 315.027 | - | | 1.1 minutes: 2%B |
| rifampicin | 822.405 | + | | 1.3 minutes: Stop |
5

## Slide 6
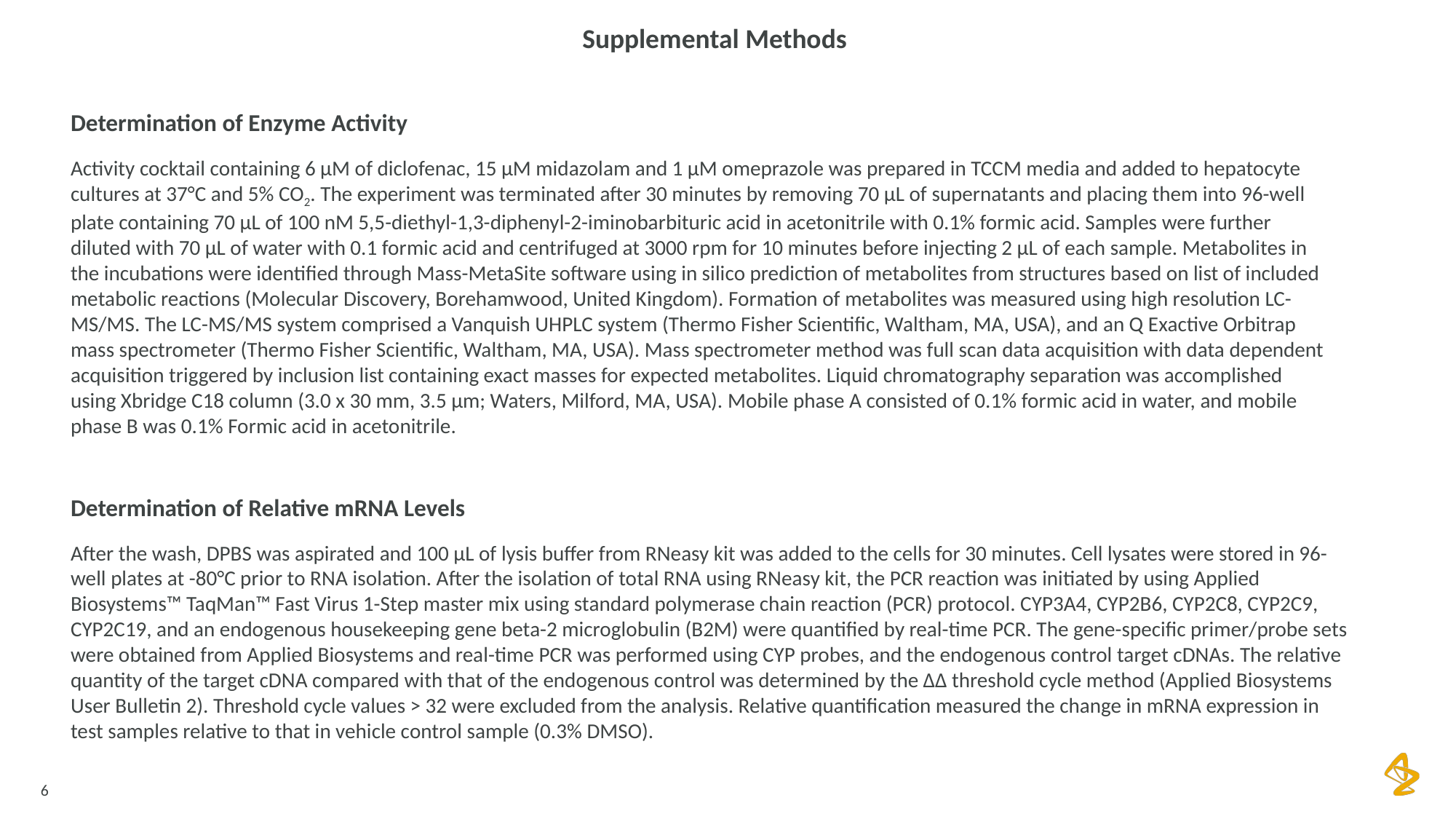

Supplemental Methods
Determination of Enzyme Activity
Activity cocktail containing 6 μM of diclofenac, 15 μM midazolam and 1 μM omeprazole was prepared in TCCM media and added to hepatocyte cultures at 37°C and 5% CO2. The experiment was terminated after 30 minutes by removing 70 μL of supernatants and placing them into 96-well plate containing 70 μL of 100 nM 5,5-diethyl-1,3-diphenyl-2-iminobarbituric acid in acetonitrile with 0.1% formic acid. Samples were further diluted with 70 μL of water with 0.1 formic acid and centrifuged at 3000 rpm for 10 minutes before injecting 2 μL of each sample. Metabolites in the incubations were identified through Mass-MetaSite software using in silico prediction of metabolites from structures based on list of included metabolic reactions (Molecular Discovery, Borehamwood, United Kingdom). Formation of metabolites was measured using high resolution LC-MS/MS. The LC-MS/MS system comprised a Vanquish UHPLC system (Thermo Fisher Scientific, Waltham, MA, USA), and an Q Exactive Orbitrap mass spectrometer (Thermo Fisher Scientific, Waltham, MA, USA). Mass spectrometer method was full scan data acquisition with data dependent acquisition triggered by inclusion list containing exact masses for expected metabolites. Liquid chromatography separation was accomplished using Xbridge C18 column (3.0 x 30 mm, 3.5 µm; Waters, Milford, MA, USA). Mobile phase A consisted of 0.1% formic acid in water, and mobile phase B was 0.1% Formic acid in acetonitrile.
Determination of Relative mRNA Levels
After the wash, DPBS was aspirated and 100 µL of lysis buffer from RNeasy kit was added to the cells for 30 minutes. Cell lysates were stored in 96-well plates at -80°C prior to RNA isolation. After the isolation of total RNA using RNeasy kit, the PCR reaction was initiated by using Applied Biosystems™ TaqMan™ Fast Virus 1-Step master mix using standard polymerase chain reaction (PCR) protocol. CYP3A4, CYP2B6, CYP2C8, CYP2C9, CYP2C19, and an endogenous housekeeping gene beta-2 microglobulin (B2M) were quantified by real-time PCR. The gene-specific primer/probe sets were obtained from Applied Biosystems and real-time PCR was performed using CYP probes, and the endogenous control target cDNAs. The relative quantity of the target cDNA compared with that of the endogenous control was determined by the ΔΔ threshold cycle method (Applied Biosystems User Bulletin 2). Threshold cycle values > 32 were excluded from the analysis. Relative quantification measured the change in mRNA expression in test samples relative to that in vehicle control sample (0.3% DMSO).
6
